# Perceived sports competence mediates the relationship between childhood motor skill proficiency and adolescent physical activity and fitness: a longitudinal assessment

**DOI:** 10.1186/1479-5868-5-40

**Published:** 2008-08-08

**Authors:** Lisa M Barnett, Philip J Morgan, Eric van Beurden, John R Beard

**Affiliations:** 1 University of Sydney, Department of Rural Health (Northern Rivers), PO Box 3074, Lismore, NSW, 2480, Australia; 2 University of Newcastle, Faculty of Education and Arts, Newcastle, NSW, Australia; 3 North Coast Area Health Service, Health Promotion Unit, Lismore, NSW, Australia; 4 Southern Cross University, Lismore, NSW, Australia; 5 New York Academy of Medicine, 1216 Fifth Ave, New York, New York, USA

## Abstract

**Background:**

The purpose of this paper was to investigate whether perceived sports competence mediates the relationship between childhood motor skill proficiency and subsequent adolescent physical activity and fitness.

**Methods:**

In 2000, children's motor skill proficiency was assessed as part of a school-based physical activity intervention. In 2006/07, participants were followed up as part of the Physical Activity and Skills Study and completed assessments for perceived sports competence (Physical Self-Perception Profile), physical activity (Adolescent Physical Activity Recall Questionnaire) and cardiorespiratory fitness (Multistage Fitness Test). Structural equation modelling techniques were used to determine whether perceived sports competence mediated between childhood object control skill proficiency (composite score of kick, catch and overhand throw), and subsequent adolescent self-reported time in moderate-to-vigorous physical activity and cardiorespiratory fitness.

**Results:**

Of 928 original intervention participants, 481 were located in 28 schools and 276 (57%) were assessed with at least one follow-up measure. Slightly more than half were female (52.4%) with a mean age of 16.4 years (range 14.2 to 18.3 yrs). Relevant assessments were completed by 250 (90.6%) students for the Physical Activity Model and 227 (82.3%) for the Fitness Model. Both hypothesised mediation models had a good fit to the observed data, with the Physical Activity Model accounting for 18% (*R*^2 ^= 0.18) of physical activity variance and the Fitness Model accounting for 30% (*R*^2 ^= 0.30) of fitness variance. Sex did not act as a moderator in either model.

**Conclusion:**

Developing a high perceived sports competence through object control skill development in childhood is important for both boys and girls in determining adolescent physical activity participation and fitness. Our findings highlight the need for interventions to target and improve the perceived sports competence of youth.

## Background

Regular participation in physical activity is associated with important short- and long-term psychological and physiological health benefits for youth [1,2]. Both physical inactivity and poor levels of physical fitness are associated with coronary heart disease risk factors in young people [3]. Physical activity dose has a strong relationship to fitness [4] and a recent review has associated cardiorespiratory fitness or endurance with both obesity and cardiovascular disease factors [5]. Yet, as children move through adolescence, physical activity participation rates [6] and cardiorespiratory fitness levels decline [7]. The identification of determinants of activity and fitness, and how these factors may interact, is therefore important in developing strategies to improve the health of youth.

Physical self-perception has been identified as an important psychosocial correlate of physical activity in youth and has been associated in cross-sectional studies with degree of participation in physical activity, (i.e. high/low, [8-11] active/non active [2,12,13]), and fitness [9,11,14]. Perceived competence, a component of physical self-perception, also correlates with physical activity behaviour [10] and with actual competence or motor skill proficiency [15,16]. Actual competence has been recently identified as an emerging correlate of youth physical activity participation [17-20] and fitness [21,22].

Perceived competence may be central to self esteem. Harter describes self esteem as a multidimensional and hierarchical construct with the self composed of different domains (i.e. social, physical, cognitive) that sit under a construct of global self esteem [23-26]. Harter's model drew from the competence or 'effectance' model of motivation which purports that behavior leads the organism to find out how the environment can be changed and what would be the consequences for the changes, leading to 'effectance' or a 'feeling of efficacy' [27]. Harter's model proposes that actual competence precedes perceived competence with perceived competence more directly effecting motivation than actual competence [23]. Others have also suggested that actual competence influences perceived competence which in turn affects physical activity participation choices [28]. It is possible that children who are skill proficient may develop a high perception of sport competence leading to greater participation in physical activity and higher fitness levels. Conversely, children with poor skill proficiency may develop low perceived competence resulting in less engagement in physical activity in adolescence. However, no longitudinal studies could be located that investigate whether physical self-perception mediates between childhood skill proficiency and subsequent physical activity behaviour and fitness in adolescence. Identification of perceived sports competence as a key mediating variable could inform the design of interventions to promote physical activity and fitness among youth.

The purpose of this paper was to investigate whether perceived sports competence mediates the relationship between childhood motor skill proficiency and (a) time spent in moderate-to-vigorous physical activity and (b) cardiorespiratory fitness levels in adolescence using structural equation modelling. This is part of a larger study known as the Physical Activity and Skills Study (PASS), a longitudinal cohort study set in New South Wales, Australia.

## Methods

### Subjects

In 2000, 1045 children from 18 randomly selected and stratified primary (elementary) schools in a study area comprising 24,555 square kilometres in New South Wales (NSW), Australia, had their proficiency in a battery of motor skills assessed for the post-test of the school-based 'Move It Groove It'(MIGI) physical activity intervention [29]. The mean age of the sample was 10.1 years (range 7.9 to 11.9 yrs). Of the students assessed at MIGI post-test, 1021 had first and last initials noted on their motor skill assessments. Of these 1021 records, 929 (91.0%) matched by full name and sex to the class roll.

In 2006/07, the list of 929 original study participants was sent to 41 consenting high schools in the original study district to identify adolescent students for follow-up as part of the PASS. One school in the study area did not consent to participation. When students' names were identified on the high school register, students were given a letter inviting them to participate, an information sheet and a consent form. Students who returned a consent form signed by their parents/guardian and themselves were included in the PASS sample.

Slightly more than half of the 928 original (one student passed away prior to consent) participants were located (51.8% n = 481/928) in 28 schools. These students were approached to participate in the study, with a consent rate of 61.7% (n = 297/481). However, 57.4% (n = 276/481) were assessed for at least one of the follow-up measures (perceived sports competence, physical activity, or fitness) as 21 consenting students could not be assessed due to school absenteeism or illness/injury. The overall follow-up rate was 29.7% (276/928). The followed up sample of 276 did not differ by sex (*χ*^2 ^= 2.40, *p *= .12) but were more likely to have been originally tested in Grade 4 (61.5%) than Grade 5 (38.5%), (*χ*^2 ^= 22.67, *p *< .0001) and had a slightly higher (17.5 compared to 16.5) mean composite childhood fundamental motor skill score (*t *= -2.60, *p *= .01).

### Data collection

Assessment of motor skills was undertaken as part of MIGI in 2000 [29]. Data on physical activity participation, cardiorespiratory endurance and perceived sports competence was collected in 2006/07. The majority of data (> 94%) were collected over Term 4 in 2006, with the remainder in Term 1, 2007 (both over summer). Order of administration was consistent for all students (weather and school priorities permitting). Cardiorespiratory endurance was assessed first (in a suitably sized indoor facility or outdoors), followed by self-reported physical activity and lastly, perceived sports competence. Data were collected by the study coordinator and three research assistants during school hours at a time convenient to the school. The assistants completed three days of training facilitated by the study coordinator and a trainer who had previously trained teachers in fundamental motor skill assessment and assessed children as part of a separate study [30]. Ethics approval was gained from the University of Sydney (07-2006/9243), the Department of Education (06.296), and the local Catholic Diocese.

### Motor skill measurement

The MIGI intervention used the Australian resource, 'Get Skilled Get Active' [31], to assess students' motor skills. An earlier version of 'Get Skilled Get Active' reported test–retest reliability of the 11 motor skills over a seven day cycle on 42 primary school children, returning reliability estimates (alpha coefficient method) of α = .70 or greater for all skills except the leap and run (α = .13 and α = .17 respectively) [32]. The updated resource included eight of the 11 skills (catch, overhand throw, kick, forehand strike, sprint run, leap, dodge, vertical jump) from the original resource [32] and four new additions (hop, side gallop, skip and static balance). Test-retest reliability has been assessed for this test battery with each grade (Grades 1–3) assessed for different combinations of six skills. Mean agreement percentage scores ranged from 69 (95% CI; 60 – 87), for the hop with Grade 1 children, to 85 (95% CI; 70–100) for the kick with Grade 3 children (Personal communication [33]).

Eight skills were assessed in 2000 using the updated protocol [31]. These skills were chosen because they are recognised as integral to the development of more sports-specific skills (e.g. development of overhand throw for the overhead smash in tennis or overhead serve in volleyball) [29]. Interrater reliability was checked on sets of 48 scores for every observer pair for all the skills combined and reported as kappa = .61 [29]. Six of these skills: three object control (kick, catch, and overhand throw), and three locomotor (hop, side gallop, and vertical jump) are reported on in this paper. A subsequent interrater reliability assessment on these six skills, using an adolescent sample, reported a weighted kappa = .70 [34].

This battery of six skills includes skills that both males and females are proficient in [35-37]. Each skill is made up of five or six features considered integral to the proficient performance of the skill. For example, the kick consists of six features: 1. Eyes focused on the ball throughout, 2. Forward and sideways swing of opposite arm, 3. Non kicking foot placed beside ball, 4. Bend knee of kick leg 90° + during backswing, 5. Contact ball with top of foot, and 6. Kick leg follows through high towards target area.

The testing procedure occurred at school either outside or in a large indoor space and allowed students to observe a motor skill demonstration before being asked to perform the skill. For the catch, kick, overhand throw and vertical jump, the skill was performed five times [38] with a feature deemed as present if the student performed it consistently throughout the trials. For the hop and side gallop, the skill was observed as students travelled back and forth once between two points 15 metres apart. The research assistant assessed each feature of that skill as present or absent without any verbal feedback from the research assistant. Assessment was completed by observing features in the order in which they are executed (also written in this order on the data sheet). For instance with the catch, 'eyes focused' is the preparatory feature, followed by 'feet moving to place the body in line with the object', then 'hands coming to meet the object' etc.

### Physical activity measurement

The Adolescent Physical Activity Recall Questionnaire (APARQ) was chosen to assess physical activity participation as it measures type of activity, frequency, duration and context of participation. The APARQ has been assessed for test-retest reliability by looking for agreement on a three category measure (vigorous, adequate, inactive) within organized or non-organized activity. Weighted kappa for Grade 10 boys and girls was reported as ranging from .44 to .89, and percentage agreement as from 67% to 97%. The APARQ was also validated against the Multistage Fitness Test [39].

Students were asked to specify all physical activities in which they participate in a usual week, in both summer and winter, and the frequency and duration of participation in each activity. Students were also asked to indicate date of birth, sex and language spoken at home.

### Cardiorespiratory fitness measure

Cardiorespiratory endurance ('fitness') was estimated indirectly as the number of laps completed on the Multistage Fitness Test (also known as the 20 meter Shuttle Run Test, Beep Test or PACER) [40]. This test was selected over other field measures of cardiorespiratory endurance such as timed and distance runs as it has been shown to be more motivational and appropriate for indoor testing, and less influenced by pacing among children and adolescents [41]. Additionally, it is considered to be an appropriate and time efficient fitness test for large groups of students [40]. Students are required to run between two lines 20 meters apart (one 'lap') starting at 8.5 km/hr and increasing by 0.5 km per hour every two minutes, in time with a recorded beep signal with each increase corresponding with a change in level. The number of 'acceptable' laps completed is determined by the student not keeping pace with the signal from the tape for two consecutive laps, (whereupon they are withdrawn from the test) or the student withdraws themselves [40].

The testing procedure involved the students being played the initial taped introduction which describes the test protocol. Briefly, students were told they must keep in time with the 'beep' and must place their foot on or over the line each time. Distance was marked out with a tape measure and students were run in groups of no more than 15 to ensure adequate spacing. Upon termination, each student had the level and shuttle written on their hand by the study coordinator and scores were recorded when all students finished.

### Physical self-perception measurement

The Physical Self-Perception Profile (PSPP) [11,42], designed to measure physical self esteem and developed from responses from college-aged American students, was selected to measure perceived sports competence. As the students in the PASS were in the upper years of high school (mean age 16 years) and close to college age, it was considered inappropriate to use the modified PSPP-CY (designed for children) [43]. The main purpose of the PSPP was to construct and validate (based on Harter's model of motivation [23-25]) a physical self-perception profile that reflected self-perception content and allowed for the hierarchical structure of self esteem [11]. There are five, six-item scales that measure perceived physical self-perception: *sports competence*, *physical condition*, *strength *and *body attractiveness *and overall *physical self-worth*. The profile uses a 4-point structured alternative format in which the student must first decide which of two statements best describes them and then must choose whether the statement is 'sort of true' or 'really true' for them. Each item can be scored from 1 (low self-perception) to 4 (high self-perception).

Fox and Corbin [11] found, through both exploratory and confirmatory factor analysis with a sample aged 19 years (mean), that all items of the PSPP contributed well to the functioning of each subscale. Corrected item total correlations for the subscale of sports competence ranged from α = .70 to .90 for females and α = .60 to .90 for males [11]. Subscales were sensitive to a wide range of individual differences, did not appear susceptible to social desirability and were stable over a three week period [11]. The sensitivity, reliability and stability of subscales were supported for both sexes [11]. The protocol recommended by Fox [42] for administering the PSPP was used in the PASS. Briefly, confidentiality was assured, and an item example was described to all students to demonstrate how to complete the survey. In order to test whether actual competence influences perceived competence, in turn affecting physical activity participation choices [28] and fitness, the subscale for sports competence was chosen for analysis (even though the PSPP was administered in its entirety).

### Data management

For the fundamental motor skill scores, the number of features rated as correct for each skill was summed for each subject. Skills with six features (all except hop and side gallop) were standardized to five and composite scores out of 15 for the three locomotor and three object control skills were constructed for use in analysis. This meant that skills with six features had greater weighting in the composite scores. A student was included if they had at least two of the required skills assessed for each composite score.

Time in physical activity by season, type of activity and activity intensity was calculated from the APARQ. Each physical activity was assigned a MET value (1 MET = 3.5 mL of oxygen per kilogram of body weight per minute) from a comprehensive list of physical activities [44], since expanded [38]. As calculated in the Schools Physical Activity and Nutrition Study [38], activities less than 10 minutes in duration, with a MET value lower than 3.0, or less than once per week, were excluded. Total time in moderate-to-vigorous activity was averaged between summer and winter. Three cases were excluded as they reported a nil value for physical activity therefore creating a skewed dataset. Time in physical activity was log transformed prior to analysis to normalize its distribution.

Scores for the Multistage Fitness Test were based on the last level and shuttle number completed by a student. This result was converted to the number of laps achieved to create a continuous variable for analysis. Cardiorespiratory fitness was square root transformed prior to analysis to normalize its distribution. The PSPP scores were summed as specified by Fox [42]. Briefly, for the subscale of perceived sports competence, scores for each item were summed with a possible range of total scores from 6 – 24. A student was included in the analysis if they had complete scores for the subscale of sports competence.

### Data analysis

Means, standard deviations and bivariate correlations were calculated for all variables. Independent *t *tests assessed cross-sectional sex differences. Two hypothesised SEMs were tested using AMOS (Versions 7.0) in SPSS (Version 15.0) to investigate whether perceived sports competence mediated the relationship between childhood skill proficiency and (a) adolescent physical activity or (b) adolescent fitness. The first model (Physical Activity Model) consisted of one latent or unobserved variable (perceived sports competence) and three measured (observed) variables (childhood object control and locomotor skill proficiency, and adolescent moderate-to-vigorous physical activity) with adolescent time in physical activity the dependent variable. The second model (Fitness Model) was structured in the same manner as the Physical Activity Model except that fitness replaced physical activity as the dependent variable.

Both models tested the possible mediating relationships between childhood object control proficiency, perceived sports competence and the outcome of interest. Object control proficiency was tested, rather than locomotor proficiency, as preliminary evidence from the PASS demonstrated that childhood object control motor skill proficiency contributes to greater adolescent physical activity participation [45] and higher fitness [46]. Others have also found that object control skill proficiency correlated with physical activity [47] and fitness [22,21]. The model thus specified a direct pathway from childhood object control skill proficiency to the dependant variable in each case, with locomotor skill proficiency included in the model only as a potential covariate of object control skill proficiency. Bivariate correlations also supported this model.

Before constructing the models, the mediating assumption [48] was tested by running separate SEMs that assessed a) the direct association by excluding perceived sports competence b) the first pathway in mediation by using perceived sports competence as the dependent variable and c) the second pathway in mediation by using perceived sports competence as the predictor variable and physical activity or fitness as the dependent variable.

Lastly, the full hypothesised structural equation models and associated regression coefficients were interpreted with reference to SEM output statistics. Perceived sports competence was modelled as a single indicator latent variable [49] in order to account for measurement error associated with this construct. For this, estimates of the random measurement error variance (calculated by multiplying the variance (*SD*^2^) of perceived sports competence by one minus Cronbachs alpha) and the regression coefficient or loading (the square root of Cronbachs alpha multiplied by the standard deviation of perceived sports competence) that associates the measured variable with its latent construct, were calculated and entered into each model. The standard deviation of perceived sports competence for both samples was 3.5 and Cronbachs alpha for perceived sports competence was .82. Thus, the random measurement error variance for perceived sports competence was 2.13 and the regression coefficient, 10.72. In SEM analyses, fit indices such as the chi-square test, Goodness of Fit Index (GFI), Tucker-Lewis fit index (or Non-Normed Fit Index (NNFI)), Comparative Fit Index (CFI), and the Root Mean Squared Error of Approximation (RMSEA) indicate whether the model fits the data. The GFI, Tucker-Lewis fit index and CFI values should be between 0 and 1, with values more than .95 considered a good fit for the data [50]. For the RMSEA, a value of less than .05 indicates a close fit [51].

The models were also tested to see if sex had a potentially moderating effect. A multi-group analysis was conducted across sex in which the fit of a constrained model (i.e. equating the variances and covariances of the measured variables across groups) was compared to an unrestrained model. A significant Δx^2 ^test between the models would indicate that a significant improvement in fit would happen if non-equivalent variances and covariances were allowed.

## Results

### Sample

Of the students followed up for the PASS, 276 completed the APARQ, 256 (92.8%) had the domain of *sports competence *calculated from the PSPP, 244 (88.4%) had fitness test results and 227 (82.3%) had all assessments completed. The Physical Activity Model used a sample of 250 and the Fitness Model used a sample of 227; both samples drawing from the potential followed up sample of 276. For both models, slightly more than half the sample were female (52.4% Physical Activity Model: n = 119/227, Fitness Model: n = 131/250) with the mean age of 16.4 years (range 14.2 to 18.3 yrs). Most were in Grade 10 in 2006/07 (Physical Activity Model 60.8% n = 150/250, Fitness Model 60.0% n = 138/230). All spoke English at home.

Descriptive statistics for both the physical activity and fitness samples (means, standard deviations and bivariate correlations) for childhood skill scores, and adolescent perceived sports competence, time in physical activity and fitness level are reported in Tables 1 and 2. In both samples, perceived sports competence in adolescence was significantly and positively associated with childhood object control skill. For the Physical Activity Model, for males and females combined, physical activity time was significantly and positively associated with perceived sports competence and object control skill. For the Fitness Model, for males and females combined, fitness was significantly and positively associated with perceived competence and object control skill. For males and females combined, locomotor skill proficiency was not significantly associated with perceived competence in either sample, or with physical activity or fitness.

**Table 1:** Physical Activity Sample: Means, Standard Deviations, and Bivariate Correlations (N = 250)

Variable	Sample	M	SD	1	2	3	4
1. Childhood Locomotor Skill (possible range 0–15)							
	Overall	8.18	3.05	_			
	Male	7.66	3.20	_			
	Female	8.65	2.83	_			
2. Childhood Object Control Skill (possible range 0–15)							
	Overall	9.54	3.20	0.22**	_		
	Male	11.36	2.67	0.36**	_		
	Female	7.88	2.71	0.38**	_		
3. Adolescent Perceived Sports Competence (possible range 6–24)							
	Overall	16.97	3.48	0.12	0.34**	_	
	Male	17.91	3.21	0.06	0.17	_	
	Female	16.11	3.51	0.26**	0.31**	_	
4. Adolescent Time in Physical Activity (minutes per week)							
	Overall	829.86	553.53	-0.08	0.28**	0.36**	_
	Male	988.9	601.42	-0.02	0.22*	0.25**	_
	Female	685.4	463.99	0.10	0.11	0.37**	_

** Correlation is significant at the 0.01 level (2-tailed)

* Correlation is significant at the 0.05 level (2-tailed)

**Table 2:** Fitness Sample: Means, Standard Deviations, and Bivariate Correlations (N = 227)

Variable	Sample	M	SD	1	2	3	4
1. Childhood Locomotor Skill (possible range 0–15)							
	Overall	8.19	3.08	-			
	Male	7.68	3.22	-			
	Female	8.66	2.89	-			
2. Childhood Object Control Skill (possible range 0–15)							
	Overall	9.67	3.20	0.26**	-		
	Male	11.52	2.65	0.39**	-		
	Female	7.99	2.69	0.46**	-		
3. Adolescent Perceived Sports Competence (possible range 6–24)							
	Overall	16.89	3.45	0.11	0.39**	-	
	Male	17.89	3.12	0.03	0.22*	-	
	Female	15.98	3.49	0.35**	0.36**	-	
4. Adolescent Fitness Level (number of laps on MFT)							
	Overall	49.83	24.04	0.03	0.39**	0.46**	-
	Male	62.40	23.55	0.02	0.11	0.23*	-
	Female	38.43	18.12	0.24**	0.23*	0.54**	-

** Correlation is significant at the 0.01 level (2-tailed)

* Correlation is significant at the 0.05 level (2-tailed)

Additionally, males were more object control proficient as children than females (Physical Activity: *t *= 10.18, *p *< .000, Fitness: *t *= 9.95, *p *< .000) whereas females were more locomotor skill proficient (Physical Activity: *t *= -2.61, *p *= .01, Fitness: *t *= -2.40, *p *= .017). As adolescents, males were more active (Physical Activity: *t *= 4.49, *p *< .000), fit (Fitness: *t *= 8.40, *p *< .000) and had higher perceived sports competence (Physical Activity *t *= 4.42, *p *< .000, Fitness: *t *= 4.32, *p *< .000), than females.

### Assumptions for mediation analysis

For the Physical Activity Model, all assumptions [48] were met, with direct relationships between: childhood object control skill proficiency and physical activity (*R*^2 ^= .08, *p *= .000), childhood object control skill proficiency and perceived sports competence (*R*^2 ^= .14, *p *< .000) and between perceived sports competence and physical activity (*R*^2 ^= .16, *p *< .000). Thus, a model to test whether perceived competence mediates between childhood object control skill and adolescent physical activity was justified. For the Fitness Model, the assumptions were also met, with a direct relationship between childhood object control skill proficiency and fitness level (*R*^2 ^= .15 *p *= .000), and between perceived sports competence and fitness level (*R*^2 ^= .26 *p *< .000). As expected, childhood locomotor skill proficiency did not predict perceived competence in either the Physical Activity Model (*p *= .07) or the Fitness Model (*p *= .08). Locomotor skill proficiency also did not predict fitness (*p *= .65) or physical activity (*p *= .90).

Thus, the hypothesised models specified direct effects from childhood object control proficiency to (a) adolescent physical activity and (b) adolescent fitness. Direct effects were also specified from childhood object control proficiency to adolescent perceived sports competence and from adolescent perceived sports competence to (a) physical activity and (b) fitness.

### Results of SEM

For both models, examination of the data revealed that univariate skewness and kurtosis was acceptable, as was multivariate kurtosis with Mardia's coefficient < 3 (Physical Activity Model -0.22, Fitness Model -2.08) [52]. The fit indices for both models also indicated a satisfactory overall fit of the hypothesised models to the data, see Table 3. Examination of the distribution of the standardized residuals for both hypothesised models reinforced the positive assessment of the models' accuracy by revealing minimal evidence of significant over or under estimation of fitted correlations with no z estimate < .01 or > .2.

**Table 3:** Results of confirmatory factor analyses for the Physical Activity and Fitness model

Sample	*n*	*x*^2^	d.f.	*P*	GFI	NNFI	CFI	RMSEA
Physical Activity Model	250	2.53	2	0.28	0.995	0.97	0.99	0.04
Fitness Model	227	2.04	2	0.36	0.996	0.98	1.0	0.01

Note: Maximum Likelihood Method was used.

Abbreviations: GFI Goodness of Fit Index; NNFI = non-normed fit index; CFI = comparative fit index; RMSEA = root mean squared error of approximation.

The models were tested to see if sex had a potentially moderating effect. For both models, the *χ*^2 ^test indicated that the data supported the structural covariance model (Physical Activity Model: *χ*^2^(10) = 11.93, *p *= .29, Fitness Model: *χ*^2^(10) = 15.01, *p *= .13). The Δ*x*^2 ^test between the unconstrained model and the nested structural covariance model for both models was also not significant (Physical Activity Model: Δ*x*^2 ^(6) = 6.93, *p *= .33, Fitness Model: Δ*x*^2 ^(6) = 7.05, *p *= .32), indicating that sex did not act as a moderator for either model.

### Prediction of adolescent physical activity time and fitness level from childhood motor skill, via perceived sports competence

For both models, childhood object control proficiency was mediated through perceived sports competence, signified by the standardized path coefficients being reduced after including perceived sports competence [48], (Physical Activity Model β = .28 compared to β = .15) and Fitness Model (β = .39 compared to β = .21). For both models, the three pathways were all significant.

**Figure 1:**
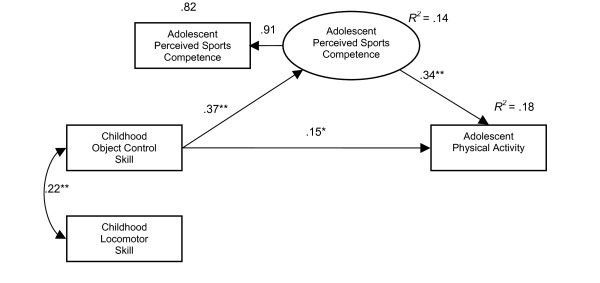
Does perceived sports competence mediate between childhood object control skill proficiency and adolescent physical activity (n = 250) *p < 0.05 **p < 0.01.

**Figure 2:**
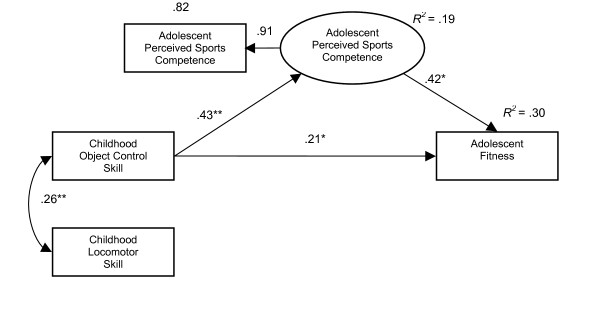
Does perceived sports competence mediate between childhood object control skill proficiency and adolescent fitness (n = 227) * p < 0.05 **p < 0.001.

The standardized indirect, direct and total effects also show that perceived sports competence acted as a mediator between childhood object control skills and both adolescent physical activity (direct β = .15, total β = .28) and fitness (direct β = .21, total β = .39), with total effects higher than direct effects, see Table 4. Also in both models, direct effects between childhood object control skill and perceived sports competence and between perceived sports competence and the dependent variables were stronger than between childhood object control skill and the dependent variables, see Figures 1 and 2.

**Table 4:** Direct, indirect and total effects (and 95% confidence intervals) of childhood object control skill on physical activity and fitness

		Childhood Object Control Skill								
		
		Direct β	LCI	UCI	Indirect β	LCI	UCI	Total β	LCI	UCI
Physical Activity Model	Time in Physical Activity	0.15	0.03	0.27	0.13	0.07	0.21	0.28	0.16	0.39
Fitness Model	Fitness Level	0.21	0.09	0.33	0.18	0.11	0.27	0.39	0.28	0.49

Abbreviations: CI = confidence intervals; β = Beta – standardized coefficients

Perceived sports competence as a mediating variable helped to explain 18% of variance of adolescent physical activity (*R*^2 ^= .18) and 30% of variance of adolescent fitness (*R*^2 ^= .30), see Figures 1 and 2. The direct relationship between object control skill proficiency and the dependent variables only explained 8% (*R*^2 ^= .08) of adolescent physical activity and 13% (*R*^2 ^= .13) of fitness variation.

## Discussion

This is the first longitudinal study to demonstrate that perceived sports competence mediates the relationship between childhood skill proficiency and adolescent physical activity or fitness. The respective structural equation models explained 18% of the variation in adolescent physical activity and 30% of the variation in adolescent fitness. Childhood locomotor proficiency did not predict perceived sports competence, physical activity or fitness. We also found that, whilst boys scored higher than girls in terms of object control proficiency, perceived competence, physical activity and fitness, sex did not moderate the relationships between these variables.

Our results suggest that being able to perform object control skills (such as catching, throwing and kicking) competently in childhood may be significant and influential in building a positive perception of sports competence, in turn increasing adolescent physical activity engagement and fitness levels. Our findings demonstrate that a positive perception of sports competence is a key predictor of physical activity and fitness outcomes and is influenced by motor skill proficiency as a child.

The strength of the relationship between object control skills and both dependent variables was almost doubled after consideration of indirect effects, indicating that perceived competence was an important mediator in each relationship. In addition, the direct relationship between childhood object control proficiency and perceived sports competence was strong in both the physical activity (β = .37) and fitness models (β = .43), signifying that adolescent perceived sports competence may be based on childhood object control ability. This shows that *being *good at object control skills as a child may help to develop a perception in adolescence that you *are *competent in sports. However, it also demonstrates that a proportion of perceived sports competence stems from other unmeasured factors. Because perception of sports competence was measured in adolescence (at the same time as physical activity and fitness), it is feasible that perception of sports competence reflects a self concept based on current skill ability, not only past (childhood) skill ability. Perception of perceived sports competence during adolescence may have therefore also been influenced by improved object control skills since measurement.

The findings support Harter's model that actual competence is a precursor to perceived competence which impacts on physical activity [23]. Other longitudinal studies have found that perception of physical condition predicted physical activity in adolescent girls (over a three year period) [53], and that motivation in physical education predicted sport and exercise participation (seven and 14 months later) [54]. Cross-sectional studies have previously demonstrated that both perceived sports competence and motor skill proficiency are important correlates of physical activity [8,10,55] and fitness [9,14,56], but were unable to determine the direction of these relationships. No longitudinal study could be located that has investigated the relationship over time between actual and perceived motor competence and physical activity and fitness.

The variance explained for fitness was higher than for physical activity, which has been found by others in cross-sectional studies. The 1997 Australian NSW Schools Fitness and Activity Survey found a battery of six motor skills explained 28% of the variance in fitness for Grade 10 girls and 18% for Grade 10 boys [22], compared to motor skill proficiency, which accounted for 8.7% of variance in children's physical activity using an objective measure [17], and 4.3% of organized activity variance in adolescents, using a self-report measure [19]. The variance explained for fitness may be greater as many physical activities (e.g. walking, swimming, riding) are not dependent on object control skill proficiency whereas many 'fitness-promoting' organised sporting activities (all ball games/sports) require a certain level of object skill proficiency for participation.

This study looked at endurance fitness only and at motor skill ability from a process-oriented perspective. But motor skills can also be assessed using product-oriented assessments, which look at skill execution such as time, distance or number of successful attempts assessed [57]. Kicking, throwing and jumping have been associated with endurance fitness using a product-oriented motor skill assessment [21]. Therefore, it may be warranted in future studies to investigate whether these aspects of motor skill fitness are predicted by object control skills.

Our study also found that boys were more proficient performing object control skills, more active, and had greater fitness levels and a higher perception of their sport competence than girls. Other studies have also shown that girls tend to perform object control skills with less proficiency [17,35-37], tend to be less fit [58] and less active than boys [59]. That childrens' and adolescents' physical self-perception differs according to sex has also been demonstrated previously [43,60], with boys scoring higher than girls on physical self-worth domains such as perceived sports competence [42,61].

However, when looking at the effect of sex on the models, there was no evidence in our study of differences between the sexes. The relationship between childhood object control proficiency and adolescent physical activity and fitness was similar for both boys and girls. Crocker [10], when looking at the relationship of PSPP domains to self-reported physical activity amongst younger adolescents, also found that there was no moderating effect for sex. It appears that adequate childhood object control skill proficiency is important for both boys and girls in developing a positive sports competence which corresponds with subsequent physical activity and fitness.

### Limitations

A study limitation is that only one third of the original matched sample was able to be followed up. This can be explained by the length of the follow-up period, the large geographic study area resulting in difficulties locating students who had moved between schools, the fact that many students of this age have legally left school, and limiting the study to schools in the original intervention area. Perhaps the greatest influence on follow-up rates is the large number of children who are likely to have left the study area over the six year study period. This should be assessed in the context of other studies for this age group which have generally had a lower consent rate [18]. There was also no differential loss to follow-up by sex, although, there was a difference in mean composite childhood skill score suggesting that followed up students may have been potentially slightly more skilled.

Another limitation of this study is the use of a self-report measure for physical activity. Whilst the APARQ has had some validation, the existing reliability and validity results are quite moderate and further validation would be beneficial [39]. Nevertheless, the APARQ was chosen for this study as it identifies and quantifies most aspects of physical activity participation and is acceptable to the target group, having been previously used in Australia in key school-based population studies [18].

## Conclusion

Our study found that perceived sports competence acted as a mediator between object control skill proficiency developed in primary school years and subsequent fitness and physical activity in adolescence. That is, for both adolescent males and females, object control skill proficiency as a child appears important in developing a positive perception of competence in sports and seems to combine to increase physical activity and fitness outcomes as an adolescent. Study strengths include a longitudinal cohort design, a valid and reliable measure of cardiorespiratory fitness, a good sample size and the use of a comprehensive battery of motor skills, equally divided between locomotor and object control. A key strategy to promote adolescent physical activity and cardiorespiratory fitness in community and school settings should be to improve perceptions of sports competence especially by targeting object control motor skill development in childhood. Teachers should be encouraged to teach motor skills using a mastery learning environment where the child is able to succeed and is encouraged to personally improve which can lead to higher levels of enjoyment and greater levels of perceived competence [62].

## Competing interests

The authors declare that they have no competing interests.

## Authors' contributions

LB participated in design of the study, data collection, statistical analysis, interpretation of data and manuscript drafting. PM, EvB and JB participated in design of the study, interpretation of data and manuscript drafting. All authors read and approved the final manuscript.
